# Molecular Dynamics Simulations to Investigate the Influences of Amino Acid Mutations on Protein Three-Dimensional Structures of Cytochrome P450 2D6.1, 2, 10, 14A, 51, and 62

**DOI:** 10.1371/journal.pone.0152946

**Published:** 2016-04-05

**Authors:** Shuichi Fukuyoshi, Masaharu Kometani, Yurie Watanabe, Masahiro Hiratsuka, Noriyuki Yamaotsu, Shuichi Hirono, Noriyoshi Manabe, Ohgi Takahashi, Akifumi Oda

**Affiliations:** 1 Institute of Medical, Pharmaceutical and Health Sciences, Kanazawa University, Kakuma-machi, Kanazawa, Ishikawa, 920–1192, Japan; 2 Graduate School of Pharmaceutical Sciences, Tohoku University, 6–3 Aoba, Aramaki, Aoba-ku, Sendai, 980–8578, Japan; 3 School of Pharmacy, Kitasato University, 5-9-1 Shirokane, Minato-ku, Tokyo, 108–8641, Japan; 4 Faculty of Pharmaceutical Sciences, Tohoku Pharmaceutical University, 4-4-1 Komatsushima, Aoba-ku, Sendai, Miyagi, 981–8558, Japan; 5 Institute for Protein Research, Osaka University, 3–2 Yamadaoka, Suita, Osaka, 565–0871, Japan; University of Akron, UNITED STATES

## Abstract

Many natural mutants of the drug metabolizing enzyme cytochrome P450 (CYP) 2D6 have been reported. Because the enzymatic activities of many mutants are different from that of the wild type, the genetic polymorphism of CYP2D6 plays an important role in drug metabolism. In this study, the molecular dynamics simulations of the wild type and mutants of CYP2D6, CYP2D6.1, 2, 10, 14A, 51, and 62 were performed, and the predictions of static and dynamic structures within them were conducted. In the mutant CYP2D6.10, 14A, and 61, dynamic properties of the F-G loop, which is one of the components of the active site access channel of CYP2D6, were different from that of the wild type. The F-G loop acted as the “hatch” of the channel, which was closed in those mutants. The structure of CYP2D6.51 was not converged by the simulation, which indicated that the three-dimensional structure of CYP2D6.51 was largely different from that of the wild type. In addition, the intramolecular interaction network of CYP2D6.10, 14A, and 61 was different from that of the wild type, and it is considered that these structural changes are the reason for the decrease or loss of enzymatic activities. On the other hand, the static and dynamic properties of CYP2D6.2, whose activity was normal, were not considerably different from those of the wild type.

## Introduction

Cytochrome P450 (CYP) is a monooxygenase that catalyzes the oxidation of several substrates [1–5]. CYPs play important roles not only in the metabolism of endogenous substrates, e.g., the biosynthesis of steroid hormones and fatty acid metabolism but also in the metabolism of exogenous materials. CYPs are known to be major drug metabolizing enzymes that can be divided into certain isoforms. CYP2D6 is one of the isoforms of drug metabolizing CYPs, and 15% of total drug metabolism in the human body is performed by CYP2D6. For example, the solubilities of several drugs are increased and the metabolites become easily excreted by CYP2D6. In addition, several active metabolites are generated from prodrugs by CYP2D6. On the other hand, CYP2D6 is the first molecular species for which genetic polymorphism has been reported, and more than 100 mutants have been found for *CYP2D6* [6–7]. The mutant genes frequently result in changes to the enzymatic reactions of CYP2D6. For example, the expression level of CYP2D6 is changed, and/or mutant proteins whose three-dimensional (3D) structures are different from that of the wild type are produced by mutant genes. The influences of mutations on enzymatic activities of CYP2D6 cause the individual differences in drug efficacies and adverse effects. Thus, the investigations on the influences of polymorphism of CYP2D6 play an important role in drug design and development. The phenotypes of CYP2D6 are classified into four types: 1) extensive metabolizer; 2) poor metabolizer; 3) intermediate metabolizer, and 4) ultra-rapid metabolizer [8]. Several important drugs, such as antidepressants and antiarrhythmic agents act as the substrate of CYP2D6, and the adverse effect of these drugs require careful control. To overcome challenges associated with polymorphism of CYP2D6, the causes of the changes of enzymatic activities for mutant CYP2D6 should be clarified. If new drugs which avoid the influences of mutations of CYP2D6 can be designed, these drugs may play important roles in tailor-made medicine. The results of a previous study suggested that P34S, F120I, and mutations of seven residues in exon 9 affect the enzymatic activities of CYP2D6 [6]. In addition, the residues Phe120, Glu216, Phe219, Glu222, Asp301, and Phe483 are considered as key residues for substrate recognition, and thus, the change of interaction patterns and/or conformational changes caused by the mutations may play important roles in enzymatic activity of mutants [9].

As mentioned above, CYP2D6 is an important enzyme within drug metabolism, and polymorphism within this enzyme requires careful attention. On the other hand, the enzymatic activities of mutant CYP2D6 were experimentally measured using several procedures, and only a few exhaustive measurements using a unified approach were reported. Moreover, although the 3D structures play important roles in the elucidation of the influence of mutations on enzymatic activities, only the 3D structures of the wild-type CYP2D6 were experimentally observed. In such cases, computational chemical methods are useful, and some structural prediction of mutant CYP2D6 was reported [6, 10–12]. Although these studies were useful for understanding the influences of mutations on CYP2D6 structures, they mainly focused on CYP2D6.17, which is the natural mutant frequently observed in the African population. For example, molecular simulations of CYP2D6.17–2, CYP2D6.17–3, CYP2D6.34, and CYP2D6.53 were conducted [11]. All the aforementioned mutants contain the mutations in the substrate recognition site (SRS). On the other hand, the 3D structures of mutants, which include mutations far from the SRS have not been sufficiently investigated. In particular, the 3D structure of CYP2D6.10, which is frequently observed in Asian populations has not been predicted. CYP2D6.10 includes the mutation P34S, which is considered as an important residue in enzymatic activity [6]. Thus, the structural predictions of the mutants, including the mutations far from the SRS appear to be useful for the investigations of the influences of polymorphism on enzymatic activities. In CYPs, the ligand access channel plays an important role in the enzymatic functions. Not only the structures but also interaction networks which form the ligand channel should be investigated.

Recently, we reported on the experimental enzymatic activities of the wild type and mutants of CYP2D6 using the unified approach [6, 10], and the activities of mutants can be compared accurately. In addition, the substrate dependencies of metabolic activities of mutants were measured using several substrates. These accurate experimental data are indispensable for clarifying the properties of CYP2D6 mutants. We conducted not only experimental but also computational studies of polymorphism of CYPs. The molecular dynamics (MD) simulations, the docking study, and MM-GB/SA calculations of CYP2D6.17 were performed for the investigations of complex formations with the inhibitor [12]. Furthermore, the structural predictions of the wild type and mutants of CYP2B6 [13] and CYP2C19 [14] were conducted. For the classical mechanical calculations of CYPs, the force field parameters for the heme iron were derived by using quantum chemical calculations of model systems [15].

In the current study, the influences of amino acid mutations on 3D structures and structural flexibilities of proteins were investigated using MD simulations of the wild type and five mutants of CYP2D6. The changes in enzymatic activities of the mutant CYP2D6 were estimated from the simulation results.

## Methods

The wild type and mutants used in the current study was shown in Table 1. Our previous studies [6, 10] reported that the enzymatic activity of CYP2D6.2 was comparable to that of the wild type for bufuralol 1’-hydroxylation. However, the activity of CYP2D6.2 for *N*-desmethyltamoxifen 4-hydroxylation was 28% of that of the wild type. These results indicated the substrate dependency of enzymatic activities for CYP2D6.2. The activity of the mutant protein CYP2D6.10 was lower than that of wild type. The mutant allele *CYP2D6*.*10* was found in 35–40% of Japanese populations [10] and was the most frequently observed allele within *CYP2D6*. The enzymatic activities of CYP2D6.14A, 51, and 62 were below the detection limit. For CYP2D6.62 including the mutation R441C, the absorption maximum of 450 nm which results from the heme iron was not experimentally observed [16]. The residue Arg441 is located near the heme in the wild type CYP2D6.1 structure. Except for the wild type CYP2D6.1, the 3D structures of mutant CYP2D6 have not as yet been experimentally observed, both for ligand-free and for ligand-bound forms.

**Table 1:** Mutation and activity of the wild-type and mutants of CYP2D6.

Protein	Mutation	Activity^*^
CYP2D6.1	Wild type	Standard
CYP2D6.2	R296C, S486T	Normal
CYP2D6.10	P34S, S486T	Decreased
CYP2D6.14A	P34S, G169R, R296C, S486T	N.D.
CYP2D6.51	R296C, E334A, S486T	N.D.
CYP2D6.62	R441C	N.D.

N.D.: not detectable

*Bufuralol 1’-hydroxylation activity [10]

For the wild type CYP2D6.1, the initial structure of the MD simulation was retrieved from Protein Data Bank (PDB) [17]. For the construction of the initial structure, the X-ray crystal structure of the complex between CYP2D6.1 and prinomastat (PDB ID: 3QM4) [18] was retrieved, and the ligand prinomastat and waters were deleted from 3QM4. The initial structures of mutants were constructed from the 3D structure of CYP2D6.1 after MD simulations by introducing the mutations.

The structural minimizations and MD simulations were performed by AMBER 12 [19]. The force field parameters for amino acid residues were ff12SB. For the heme, our determined parameters for the heme iron [15], and Giammona parameters were used. Our parameters represented the sextet state with five-coordinate iron (III), and the spin and coordination states of CYP2D6 were the same as those described in a previous study [15]. The TIP3P water solvent model with a thickness of at least 8 Å was used, and sodium ions were added as counterions. The system, including an explicit solvent model, was simulated with a periodic boundary condition, and the particle mesh Ewald method [20] was used to process long-range electrostatic interactions. A cutoff of 10 Å was used for nonbonding interactions. The complements of the hydrogen atoms which could not be observed by X-ray crystallographical analysis and the artificially mutated residues were performed by the tleap module of AmberTools. First, the structural minimizations were conducted for the initial structures. The 1000-cycle minimizations of water molecules and counterions were conducted, followed by the 1000-cycle minimizations of the whole system. After the minimizations, temperature-increase MD simulations to 300 K with the fixed protein were performed following which MD simulations at 300 K were performed. The 20 ps simulations were performed for the temperature-increase of MD and 250 ns simulations were conducted for the equilibration MD. The covalent bonds containing hydrogen atoms were restricted by SHAKE [21]. The 3D structures obtained by equilibration MD simulations were minimized, and the minimized structures were used for the analysis. In addition, the MD simulation of CYP2D6.1 using ff14SB force field was also performed, and the result was compared with the result of MD simulation using ff12SB force field. The calculations using ff14SB force field were conducted by AMBER14.

To assess the convergence of the MD simulations, root mean square deviations (RMSDs) were calculated. The initial structures of MD were used as the reference structures of RMSD calculations. In addition, root mean square fluctuations (RMSFs) were calculated to evaluate the structural flexibilities of proteins. The RMSFs were calculated using the final 20 ns trajectories of equilibration MD simulations, and the average structures of the final 20 ns trajectories were used as the reference structures of RMSF calculations. In addition, the occurrence rates of hydrogen bonds and the interatomic distances were calculated using the final 20 ns trajectories. These analyses were performed using the cpptraj module of AmberTools 12.

## Results and Discussion

**Fig 1:**
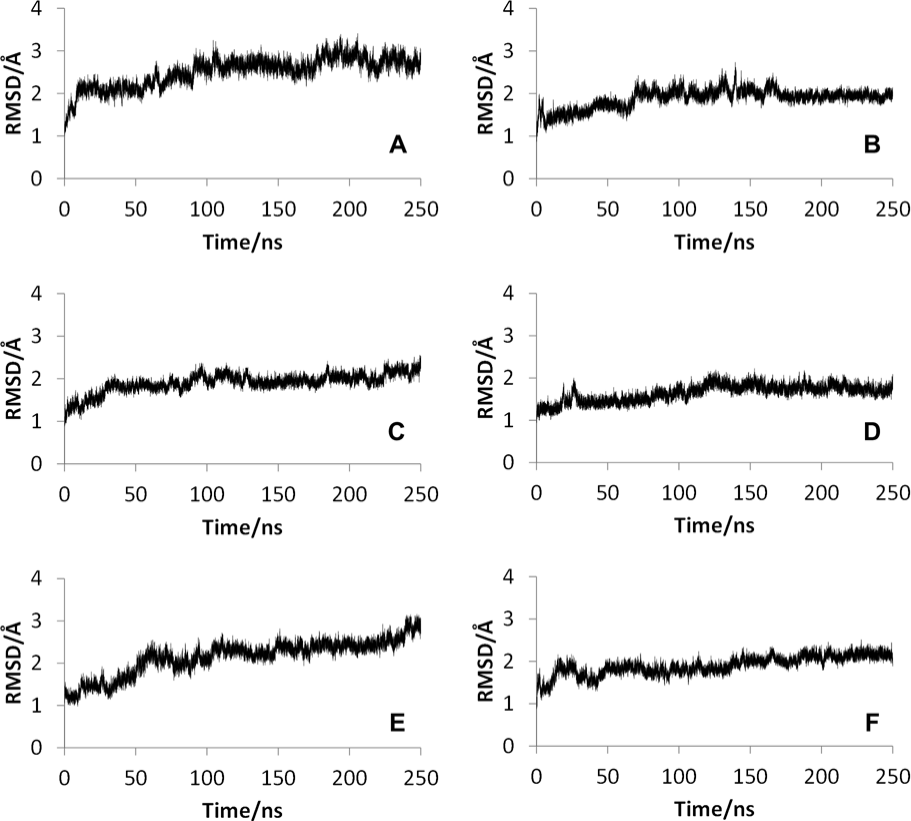
Root mean square deviations (RMSDs) for the wild type and mutants of CYP2D6. **(A) CYP2D6.1, (B) CYP2D6.2, (C) CYP2D6.10, (D) CYP2D6.14A, (E) CYP2D6.51, (F) CYP2D6.62.** The reference structures of RMSD calculations were the initial structures of molecular dynamics simulations. Thus, the reference structure for CYP2D6.1 was the minimized crystal structure. For the mutants, initial structures constructed from three-dimensional structures of CYP2D6.1 were used as the reference.

The RMSDs for the wild type and the mutants of CYP2D6 are shown in Fig 1. As shown in Fig 1, simulations for the wild type and all mutants except for CYP2D6.51 converged. Because the RMSDs except for that for CYP2D6.51 converged in the final 20 ns of simulations, the analyses described below were performed using the final 20 ns trajectories. The results indicated that the equilibrium structures can be obtained by the extended MD simulations for these wild types and mutants except for CYP2D6.51. On the other hand, the MD simulation of CYP2D6.51 did not converge, even though 250 ns simulations were performed. In addition, the averaged RMSD value of the final 1 ns structures of the MD simulation was 2.86 Å for CYP2D6.51, although those for CYP2D6.2, CYP2D6.10, CYP2D6.14A, and CYP2D6.62 were approximately 1.81 ~ 2.34 Å, indicating that the 3D structure of CYP2D6.51 was largely changed from that of the initial structure in comparison with 3D structures of other mutants. Because the initial structures were constructed from the 3D structure of the wild type CYP2D6.1, the 3D structure of CYP2D6.51 was largely different from that of CYP2D6.1. The enzymatic activity of CYP2D6.51 was below the detection limit, and the loss of activity may have been caused by the large structural change of CYP2D6.51.

**Fig 2:**
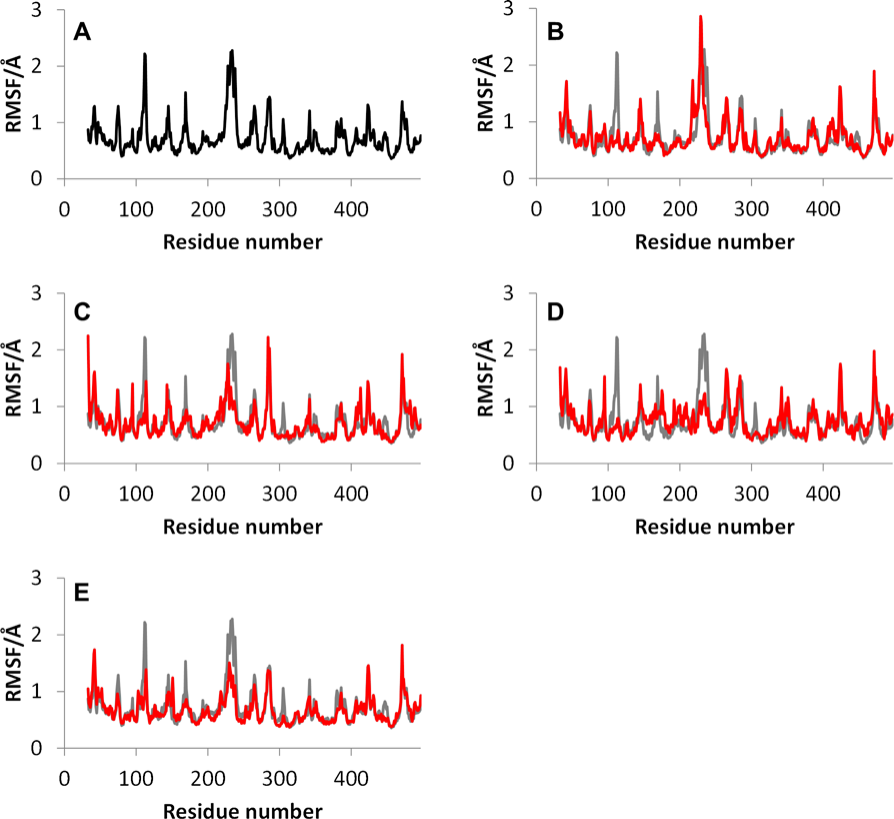
Root mean square fluctuations (RMSFs) for the wild type and mutants of CYP2D6. **(A) CYP2D6.1, (B) CYP2D6.2, (C) CYP2D6.10, (D) CYP2D6.14A, (E) CYP2D6.62.** The simulation of CYP2D6.51 did not converge. For the mutant CYP2D6, RMSF values (red) were compared with those of the wild type (gray).

The RMSFs of the wild type and mutants without CYP2D6.51, whose MD simulations did not converge, are shown in Fig 2. The RMSF values were calculated using the final 20 ns trajectories. In Fig 2A, only the RMSF values of the wild type CYP2D6.1 are shown. In other figures, RMSF values of the mutants are illustrated with those of CYP2D6.1 for comparison. For the RMSFs of CYP2D6.1, the high peaks were observed in the B-C loop (near the 112th residue) and F-G loop (near the 230th residue). These peaks indicated that these portions of the CYP2D6.1 are flexible. The F-G loop is adjacent to B-C loop, and F-G loop is considered to be associated with the formation of the substrate access channel [22]. As mentioned above, wide varieties of substrates are known to be metabolized by CYP2D6. The flexibility of the F-G loop, which is a part of the substrate access channel, may cause the substrate diversity of CYP2D6.

**Fig 3:**
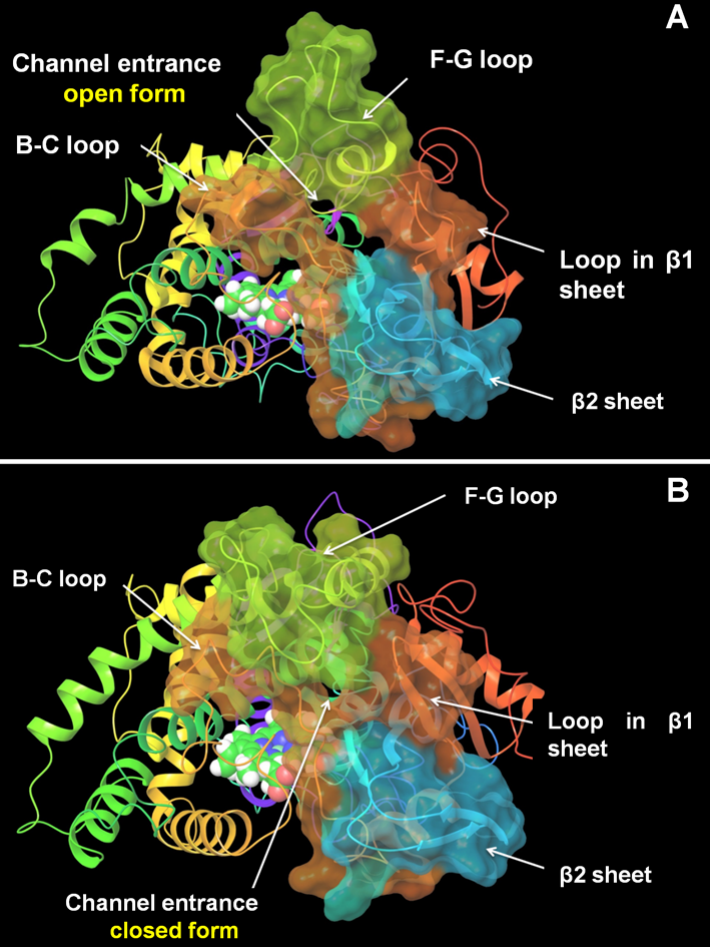
Channel entrance of CYP2D6. **(A) CYP2D6.1 (B) CYP2D6.10.** The three-dimensional structure of CYP2D6.1 obtained by the molecular dynamics simulation at 150 ns was of the open form, whereas that of CYP2D6.10 was of the closed form.

As shown in the RMSFs of mutants, the flexibility of the F-G loop was maintained only in CYP2D6.2 and the RMSF values of CYP2D6.10, CYP2D6.14A, and CYP2D6.62 at F-G loops were approximately 0.5 Å (or greater) lower than that of the wild type CYP2D6.1. In addition, narrow peaks for CYP2D6.10 and CYP2D6.62 were observed at 228th and 226th residues, respectively. Because the broad peak was observed for CYP2D6.1 at 230th residue, the flexibilities of F-G loop regions for CYP2D6.10 and CYP2D6.62 were different from that for CYP2D6.1. The 3D structure of CYP2D6.10, in which the flexibility of the F-G loop was lower than that of CYP2D6.1, was compared with the structure of CYP2D6.1. The structures proximate the substrate access channel for CYP2D6.1 and CYP2D6.10 obtained by the MD simulations are illustrated in Fig 3. Because the structural fluctuation was observed for CYP2D6.1 as mentioned below, calculated structures at 150 ns are shown in these figures. The F-G loop was considered as the “hatch” of the channel. As shown in the 3D structure of CYP2D6.1 in Fig 3A, the entrance of the substrate access channel was constructed from the F-G loop, the loop in the β1 sheet, β2 sheet, and B-C loop. In this structure of CYP2D6.1, the “hatch” F-G loop was open. On the other hand, for CYP2D6.10, the substrate access channel was of the closed form as the F-G loop was closed, as shown in Fig 3B. In addition to the closed form of CYP2D6.10, the opening of the hatch of the channel by CYP2D6.10 appears to be difficult, even if the substrate approaches the channel entrance, because of the low flexibility of the F-G loop. Because the substrate cannot enter into the proteins when the channel entrance is closed, the 3D structure and the structural flexibility of the channel entrance appears to be the reason for the low enzymatic activity of CYP2D6.10.

**Fig 4:**
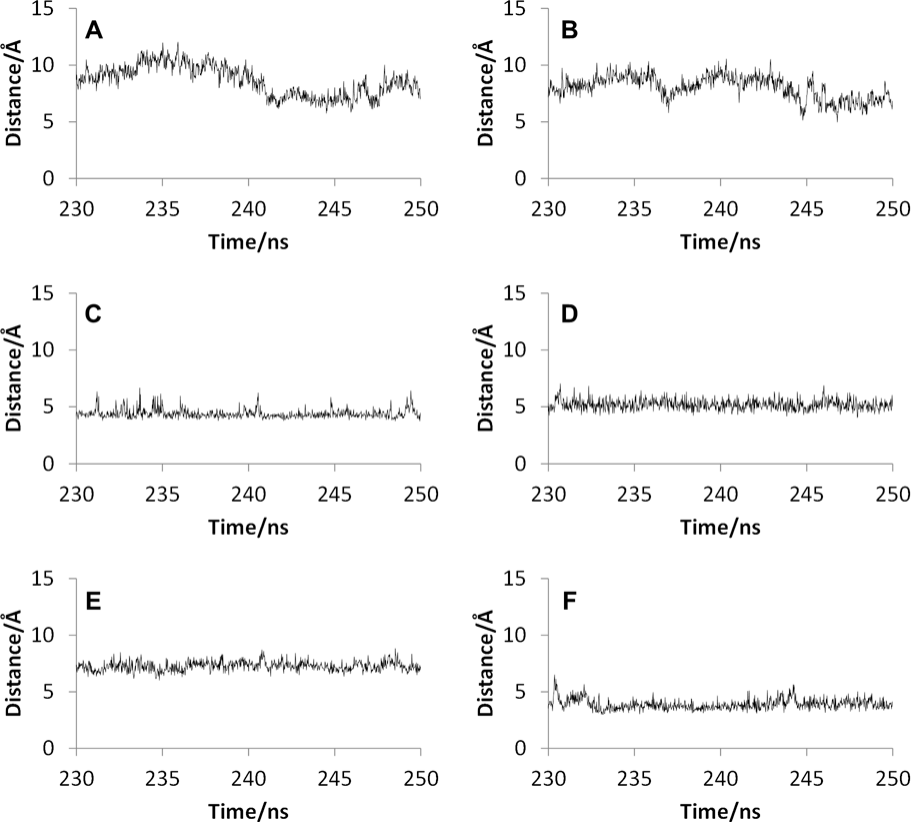
Distance between the B-C and the F-G loop. **The closest amino acid pairs were different for different mutants.** The distances between (A) Val104 and Asn225 in CYP2D6.1, (B) Val104 and Val227 in CYP2D6.2, (C) Ile109 and Leu236 in CYP2D6.10, (D) Ile109 and His232 in CYP2D6.14A, (E) Leu110 and Leu241 in CYP2D6.51, and (F) Pro105 and Val229 in CYP2D6.62 are shown.

To clarify the positional relationship between the F-G and the B-C loops which formed the channel entrance, the distance between the F-G and the B-C loops in the final 20 ns trajectories of the MD simulations are shown in Fig 4. For both the wild type and the mutants, the distance between the nearest neighboring Cα atoms in the F-G and the B-C loops were calculated. In S1 Fig of Supporting Information, the residues used for the distance calculations are illustrated. As shown in S1 Fig, the nearest neighboring residues in the F-G and the B-C loops were different for different mutants, therefore, the different residues were used for the distance calculations of different mutants. As shown in Fig 4, although the averaged distances for CYP2D6.1 in the final 20 ns trajectory were 8.62 Å, the distances for CYP2D6.10 were only 4.38 Å. These results indicated that the channel entrance of CYP2D6.10 is of the closed form, and the entrance is difficult to open and is maintained in the closed position because of the low flexibility of the F-G loop.

**Fig 5:**
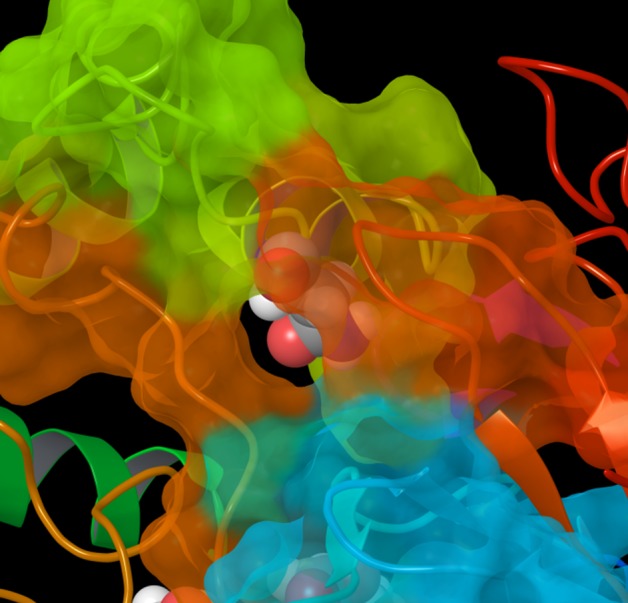
Channel entrance for CYP2D6.2. Asn225 is illustrated in space fill model.

For CYP2D6.2, the structural flexibility of the F-G loop was not lower than that of CYP2D6.1. In fact, the RMSF value of the F-G loop was more flexible than that of the wild type, as shown in Fig 2B. On the other hand, as shown in Fig 4B, the distance between the F-G and the B-C loops were shorter than that of CYP2D6.1. For CYP2D6.1, both the long and short distance values were observed. Thus, for CYP2D6.2, not only the average distance but also the range of distance was shorter than those for CYP2D6.1.The results indicate that the substrate access channel was closed for CYP2D6.2, in comparison with CYP2D6.1. However, because the distance was longer than that of CYP2D6.10, the hatch of the channel entrance was not completely closed. In addition, the residues of channel entrance for CYP2D6.2 were similar to CYP2D6.1, as shown in S1 Fig. Thus, the substrate may occasionally enter into the active site of CYP2D6.2. In addition, the distances were occasionally longer than 10 Å during the final 20 ns trajectory for CYP2D6.2 as shown in Fig 4B, indicating that the hatch of the channel entrance randomly opened and closed similar to that of CYP2D6.1. For CYP2D6.1 and CYP2D6.2, the distances were longer than 10 Å in 17.8% and 1.1% of final 20 ns trajectories, respectively. These results indicate that the hatch of CYP2D6.1 was opened more frequently than that of CYP2D6.2. The opening-and-closing behavior of the hatch may be caused by the high flexibility of the F-G loop as shown in Fig 2. The substrate specificity of CYP2D6.2 is known to be different from that of CYP2D6.1. The semi-closed channel entrance and low frequency of opening-and-closing behavior of the hatch may influence the substrate specificity. Fig 5 illustrates Asn225 located near the entrance of the substrate access channel in CYP2D6.2. Because the substrates of CYP2D6 are frequently known to include the basic nitrogen and the aromatic ring [3], the hydrophilic amino acid residue Asn225 may play a particular role in the substrate selections of CYP2D6 by interacting with basic nitrogen. Asn225 was located near the channel entrance only for CYP2D6.1, CYP2D6.2, and CYP2D6.14A, and was buried in the protein interior for CYP2D6.10 and CYP2D6.62. Asn225 might act as a “gatekeeper” of the channel, and may affect the substrate specificity of CYP2D6.

**Fig 6:**
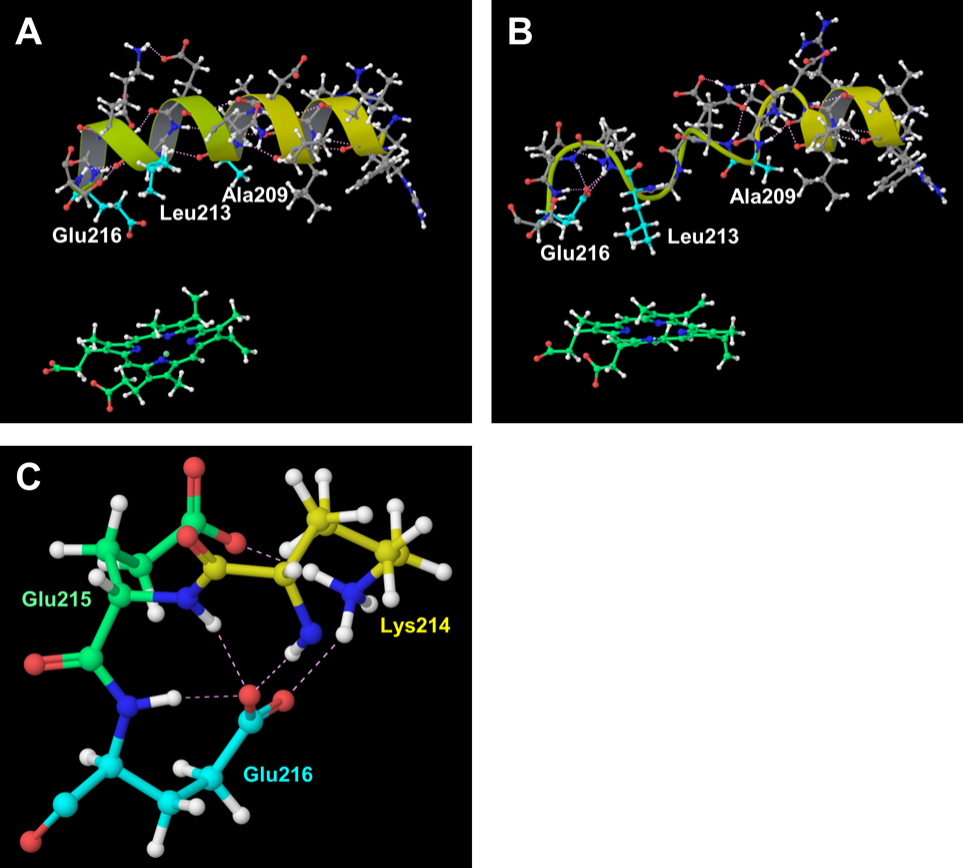
**F helix. (A) CYP2D6.1, (B) CYP2D6.14A, and (C) hydrogen bonds proximate Glu216 in CYP2D6.14A.** The hydrogen bonding network and secondary structures of CYP2D6.14A were different from those of the wild type CYP2D6.1.

For CYP2D6.14A, the flexibility of the F-G loop was lower than that of CYP2D6.1 as shown in Fig 2D. In addition, the distance between the F-G and the B-C loops was short. These results were similar to those of CYP2D6.10. Thus, these results indicate that the substrate access channel is of the closed form and CYP2D6.14A finds it difficult to open the channel entrance. In addition, the 3D structure proximate the F helix in CYP2D6.14A was different from that of CYP2D6.1 (Fig 6). Part of the F helix acts as the SRS [23–24]. As reported in a recent study [18], the large conformational changes of the side chain of the residues in and around the F helix, i.e., Ala209, Leu213, and Glu216, were caused by the substrate binding to CYP2D6. These conformational changes appear to affect the shape and size of the active site. In addition, Glu216 was reported to form the hydrogen bond with the substrate, and it is regarded as the key residue for substrate recognition [9, 25]. In our computational result for CYP2D6.1, the F helix was constructed from the 202th–216th residues and was located near and above the heme. On the other hand, the F helix included only 202th–206th residues in CYP2D6.14A, and the hydrogen bonding network was largely broken in CYP2D6.14A in comparison with CYP2D6.1. The conformations of not only the main chain but additionally the side chain in CYP2D6.14A were different from those of CYP2D6.1. The side chain of Leu213 approached the heme and broke into the pocket above the heme. The side chain carboxyl group of Glu216 turned away from the heme. These results indicate that the 3D structure and the interaction network in the substrate recognition pocket are influenced by the mutations of residues. The changes of the 3D structures and interaction patterns may cause the loss of enzymatic activity. Furthermore, Glu216 formed the hydrogen bonds with adjacent residues in CYP2D6.14A, although these hydrogen bonds were not observed in CYP2D6.1. Fig 6C illustrates these hydrogen bonds in CYP2D6.14A. The occurrence rates of the hydrogen bonds related to the side chain carboxyl group of Glu216 in CYP2D6.14A are shown in Table 2. As shown in Table 2, the occurrence rates of the hydrogen bonds between the carboxyl group of Glu216 and the adjacent residues were high. Because the carboxyl group of Glu216 formed the intramolecular hydrogen bonds, the intermolecular hydrogen bonds between Glu216 and the substrate may not be formed in CYP2D6.14A. The lack of the intermolecular hydrogen bonds influences substrate recognition, and therefore, the intramolecular hydrogen bonds may be the cause of the loss of the enzymatic activity of CYP2D6.14A.

**Table 2:** Occurrence rates of hydrogen bonds found proximate Glu216 in CYP2D6.14A.

Hydrogen bond	Occurrence rate
Glu216@Oε1-Lys214@N	98%
Glu216@Oε1-Glu215@N	93%
Glu216@Oε1-Glu216@N	73%
Glu216@Oε2-Lys214@Nζ (Hζ1)	32%
Glu216@Oε2-Lys214@Nζ (Hζ2)	32%

**Fig 7:**
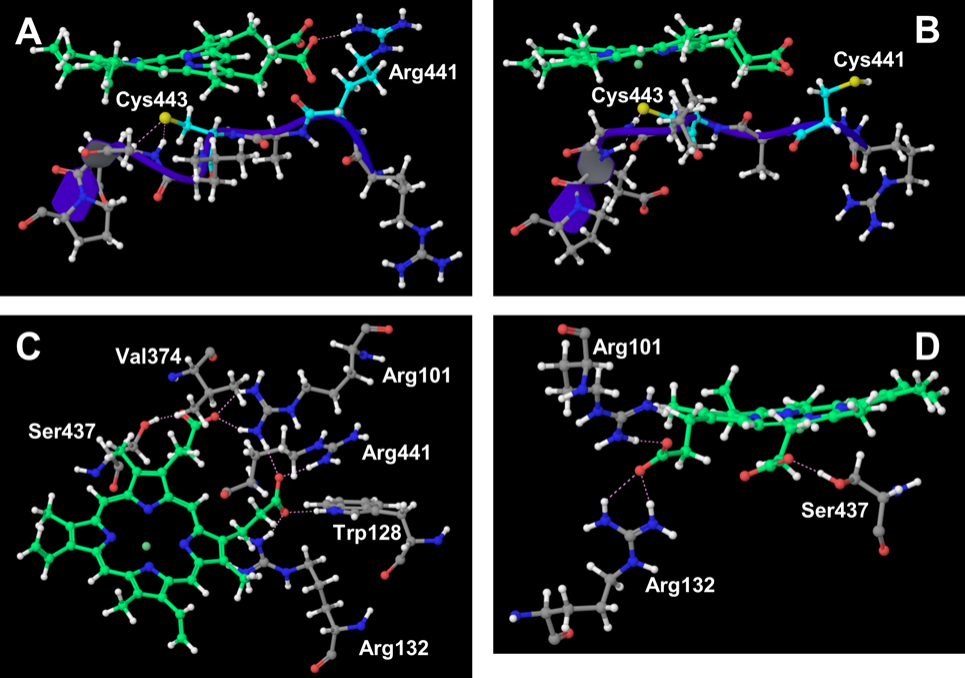
Structures proximate the heme for CYP2D6.1 and CYP2D6.62. (A) Ionic interactions between Arg441 and the heme in CYP2D6.1, (B) neither ionic bond nor hydrogen bond between Cys441 and the heme in CYP2D6.62, (C) hydrogen bonds proximate the heme in CYP2D6.1, and (D) hydrogen bonds proximate the heme in CYP2D6.62. In the static structure of CYP2D6.1 at 250 ns, the hydrogen bond between Val374 and heme is not appeared (Fig C).

In addition, for CYP2D6.62, the flexibility of the F-G loop was lower than that of the wild type, similar to CYP2D6.10 and CYP2D6.14A (Fig 2(E). Furthermore, the distance between the F-G and the B-C loop was short. These results indicated that the hatch of the substrate access channel was closed also for CYP2D6.62. Furthermore, the static structure of CYP2D6.62 was different from that of CYP2D6.1. In the current study, the heme moiety was included in CYP2D6.62, and the MD simulation was performed for the holo protein. The MD simulation showed that the interaction mode around the heme was different from that of CYP2D6.1. The 3D structures proximate the heme for CYP2D6.1 and CYP2D6.62 are shown in Fig 7. The mutation R441C for CYP2D6.62 was located near Cys443, which is the vertical ligand of the heme iron. For the wild type CYP2D6.1, the interionic electrostatic interaction between Arg441 and the carboxyl group of the porphyrin ring of the heme was observed. On the other hand, because the Arg441 mutated into cysteine, the interionic interaction and/or the hydrogen bond was not observed between the 441th residue and the heme. Tables 3 and 4 illustrate the occurrence rates of the hydrogen bonds (including interionic electrostatic interactions) between the carboxyl group of the porphyrin ring of the heme and amino acid residues for CYP2D6.1 and CYP2D6.62, respectively. The occurrence rates were calculated by using the final 20 ns trajectories. In CYP2D6.1, the interactions were observed for Arg101 (three interactions), Trp128, Arg132, Val374, Ser437, and Arg441. Thus, a total of eight interactions were formed for CYP2D6.1. On the other hand, the interactions were observed for Arg132 (two interactions), Arg101, and Ser437; therefore, a total of four interactions were formed. The results indicated that the heme is not strongly recognized by CYP2D6.62 in comparison with CYP2D6.1 as the interactions between the heme and the protein for CYP2D6.62 were weaker than that for CYP2D6.1. Thus, CYP2D6.62 may lack the heme. The computational results were consistent with the experimental results in which the absorption maximum of 450 nm attributed to the heme iron was not observed for CYP2D6.62 [16]. The absence of the heme appears to be the reason of the loss of the enzymatic activity of CYP2D6.62.

**Table 3:** Occurrence rates of hydrogen bonds including the heme in CYP2D6.1.

Hydrogen bond	Occurrence rate
HEM@O1-Arg441@Nη	95%
HEM@O1-Arg101@Nη1 (Hη1)	93%
HEM@O2-Arg101@Nη1 (Hη2)	92%
HEM@O3-Arg132@Nη	89%
HEM@O3-Trp128@Nε	75%
HEM@O4-Ser437@Oγ	69%
HEM@O2-Arg101@Nη2	55%
HEM@O4-Val374@N	10%

**Table 4:** Occurrence rates of hydrogen bonds including the heme in CYP2D6.62.

Hydrogen bond	Occurrence rate
HEM@O1-Ser437@Oγ	95%
HEM@O2-Arg101@Nη	93%
HEM@O3-Arg132@Nη1	72%
HEM@O3-Arg132@Nη2	12%

**Fig 8:**
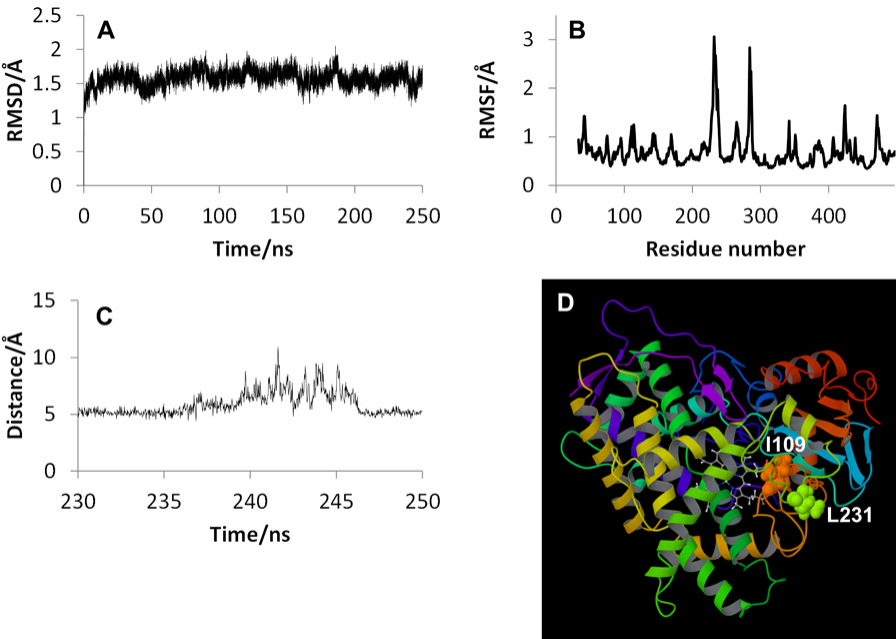
Results of MD simulation for CYP2D6.1 using ff14SB force field. (A) RMSD, (B) RMSF, (C) distance between B-C and F-G loops, and (D) entrance residues used for the distance calculation.

In Fig 8, the results of MD simulation for CYP2D6.1 using ff14SB force field for amino acid residues are illustrated. RMSD (Fig 8A) and RMSF (Fig 8B) were calculated by same procedures as Figs 1 and 2. As shown in these figures, the MD simulation using ff14SB was also converged and the F-G loop was highly flexible, similar to the results of the ff12SB simulation. For the RMSF shown in Fig 8B, the peak located in B-C loop was lower than that of H-I loop (near the 284th residue). The peak of the H-I loop was observed also in Fig 2A, and it was the fourth-highest peak in Fig 2A. In addition, the RMSF data of 150 ns simulation using ff12SB was similar to the data shown in Fig 8B, and the results of the MD simulations using both ff12SB and ff14SB indicate that the flexibility of the F-G loop is the highest of all regions in CYP2D6.1. The distance between the B-C and the F-G loops shown in Fig 8C was calculated by using two residues shown in Fig 8D. The distance indicates the opening-and-closing behavior of the hatch. These results suggest that the structural features of CYP2D6 can be calculated by MD simulations with both ff12SB and ff14SB force fields.

## Conclusion

**Fig 9:**
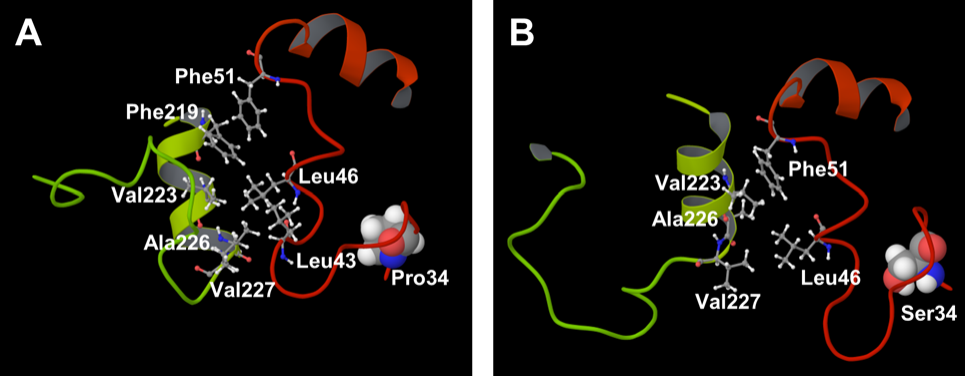
3D structures of the N-terminal region (red-orange) and the F-G loop region (yellow-yellow green). (A) CYP2D6.1, (B) CYP2D6.10. The 34th residues are shown in space fill model, and the hydrophobic residues located in the interdomain regions are illustrated in ball-and-stick model.

In the current study, the static and dynamic properties of the wild type and the five mutants of CYP2D6 were investigated by the MD simulations. The enzymatic activities of four of the mutants were lower than that of the wild type, and the reasons for the low activities were estimated. The results of the MD simulations indicated the importance of the structure and the motion of the hatch of the substrate access channel. In addition, the lack of the heme moiety may cause the change of the interaction modes resulting from the mutation. For CYP2D6.62, the structural changes near the mutated residue (R441C) appear to be important in the enzymatic activity. On the other hand, for CYP2D6.10, the structural changes of the F-G loop, which was distant from the mutated residues (P34S, S486T), appear to be important. The result suggests that the amino acid mutations influence even the distant residue from the mutated position. In CYP2D6.10, the mutation P34S influenced the hydrogen bonding network, and the change of the network affected the 3D structure of the F-G loop which was located far from 34th residue. The 3D structure of the N-terminal loop and the F-G loop is illustrated in Fig 9. The hydrophobic residues located in the interdomain region are shown in this figure. As shown in the figure, hydrophobic residues, i.e., Leu43, Leu46, Phe51, Phe219, Val223, Ala226 and Val227 were located in the interdomain region in CYP2D6.1, and the hydrophobic effect seems to play an important role in the interaction between the N-terminal region and the F-G loop. On the other hand, in CYP2D6.10, Leu46, Phe51, Val223, Ala226 and Val227 were located in the interdomain region. Because the number of hydrophobic residues in interdomain region between the N-terminal and the F-G loops was fewer in CYP2D6.10 than that in CYP2D6.1, the difference of hydrophobic effects may affect the 3D structure of the F-G loop. That is, the 3D structure and property of the N-terminal loop was changed by the mutation P34S, and the changes of the structure and property of the N-terminal loop influenced the hydrophobic contacts in interdomain region. The change of hydrophobic contacts may change the 3D structure of the F-G loop. As shown in Fig 9, the influences of the mutation P34S may spread from the N-terminal loop to the distant portion of the protein through the hydrophobic effects.

By using the MD simulations, the structural prediction of structurally unknown proteins is possible. For the mutants of drug metabolizing enzymes in particular, a large number of the natural mutants were generally found. For example, more than 100 mutants were detected for CYP2D6. Thus, the experimental observations of all mutants are not practical, and the computational procedures can efficiently support the experiments. In addition, the MD simulations can reveal the dynamic properties of proteins, such as structural flexibilities. The MD simulations are expected to be useful for the structural biological studies for the polymorphism of the drug metabolizing enzymes.

## Supporting Information

S1 Fig Entrance residues of CYP2D6. The distances between these two residues are calculated, and the results are illustrated in Fig 4. (A) CYP2D6.1, (B) CYP2D6.2, (C) CYP2D6.10, (D) CYP2D6.14A, (E) CYP2D6.51, (F) CYP2D6.62.
(PDF)
