# Supplementary material for: Molecular Dynamics Simulations to Investigate the Influences of Amino Acid Mutations on Protein Three-Dimensional Structures of Cytochrome P450 2D6.1, 2, 10, 14A, 51, and 62
Source: PLoS One. 2016 Apr 5;11(4):e0152946. doi: 10.1371/journal.pone.0152946 (PMC4821567; doi:10.1371/journal.pone.0152946)
Supplement: S1 Fig — The distances between these two residues are calculated, and the results are illustrated in Fig 4. (A) CYP2D6.1, (B) CYP2D6.2, (C) CYP2D6.10, (D) CYP2D6.14A, (E) CYP2D6.51, (F) CYP2D6.62. (PDF) [file pone.0152946.s001.pdf]

Supporting Information

Molecular dynamics simulations to investigate the influences of amino acid mutations on protein three-dimensional structures of cytochrome P450 2D6.1, 2, 10, 14A, 51, and 62

Shuichi Fukuyoshi[1†], Masaharu Kometani[1†], Yurie Watanabe[1], Masahiro Hiratsuka[2], Noriyuki Yamaotsu[3], Shuichi Hirono[3], Noriyoshi Manabe[4], Ohgi Takahashi[4], Akifumi Oda[1, 5*]

[1]Institute of Medical, Pharmaceutical and Health Sciences, Kanazawa University, Kakuma-machi, Kanazawa, Ishikawa 920-1192, Japan

[2]Graduate School of Pharmaceutical Sciences, Tohoku University, 6-3 Aoba, Aramaki, Aoba-ku, Sendai 980-8578, Japan.

[3]School of Pharmacy, Kitasato University, 5-9-1 Shirokane, Minato-ku, Tokyo 108-8641, Japan

[4]Faculty of Pharmaceutical Sciences, Tohoku Pharmaceutical University, 4-4-1 Komatsushima, Aoba-ku, Sendai, Miyagi 981-8558, Japan

[5]Institute for Protein Research, Osaka University, 3-2 Yamadaoka, Suita, Osaka 565-0871, Japan

[*]E-mail: oda@p.kanazawa-u.ac.jp

[†]These authors contributed equally to this work.

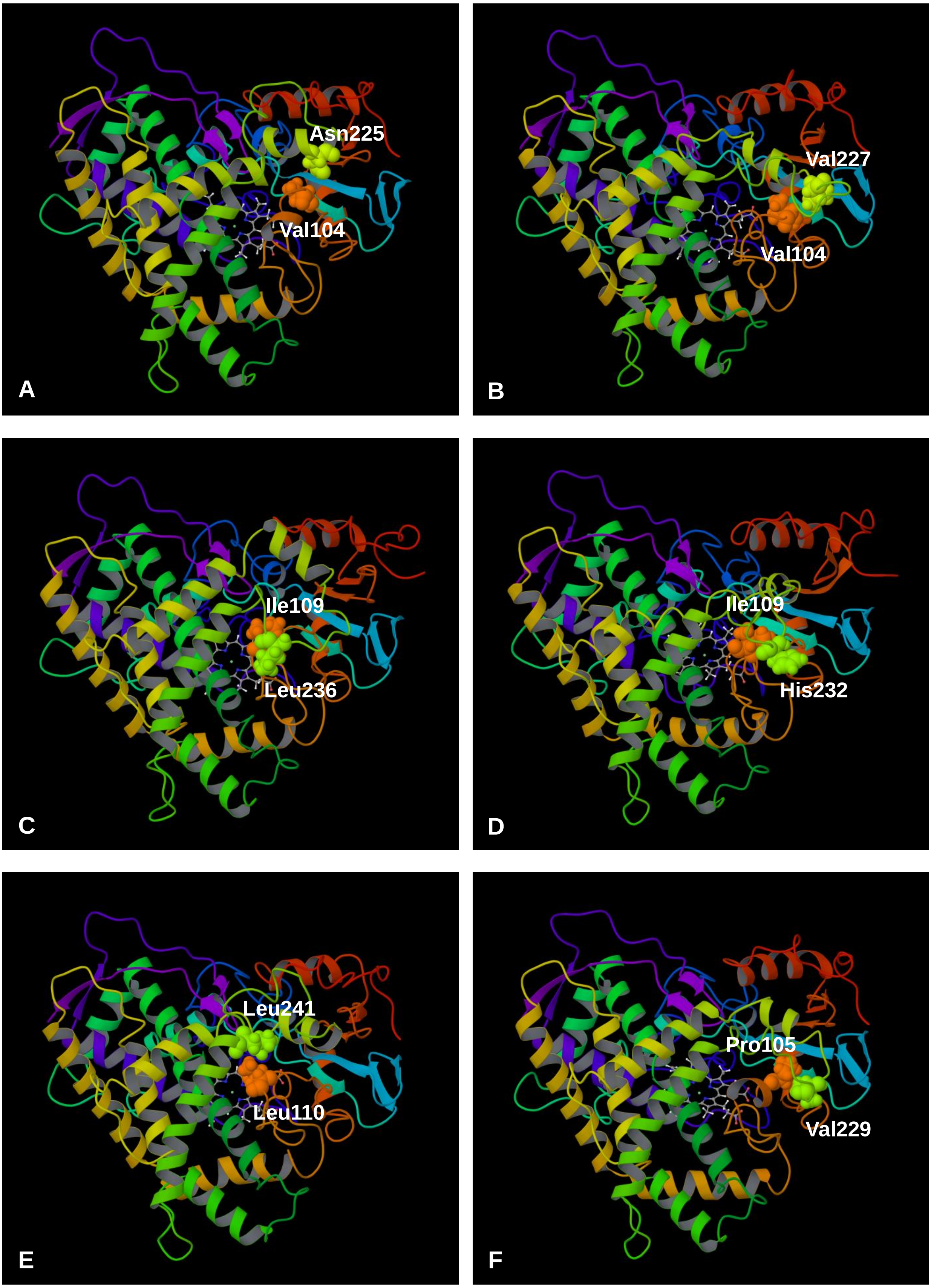

S1 Fig. Entrance residues of CYP2D6. The distances between these two residues are calculated, and the results are illustrated in Figure 4. (A) CYP2D6.1, (B) CYP2D6.2, (C) CYP2D6.10, (D) CYP2D6.14A, (E) CYP2D6.51, (F) CYP2D6.62.
